# Monitoring of virological response and acquired HIV-1 drug resistance among patients initiating antiretroviral therapy in the Centre Region of Cameroon

**DOI:** 10.11604/pamj.2025.52.122.46773

**Published:** 2025-11-21

**Authors:** Bello Djoda, Jerome Fru-Cho, Patrick Achiangia Njukeng, Ezechiel Ngoufack Jagni Semengue, Patrick Valere Tsouh Fokou, Evariste Molimbou, Naomi-Karell Etame, Rodrigue Kamga Wouambo, Bauer Wilhelm, Christopher Dächert, Alain Nantchouang Megaptche, Maximilian Muenchhoff, Abel Wade, Stefanie Van Cleaf, Josef Eberle, Charles Fokounang, Joseph Fokam, Oliver Till Keppler, Oliver Bosnjak

**Affiliations:** 1 Department of Microbiology and Parasitology, Faculty of Science, University of Buea, Buea, Cameroon,; 2 Max von Pettenkofer Institute and Gene Center, Virology, National Reference Center for Retroviruses, Faculty of Medicine, Ludwig-Maximilians-Universität München, LMU Munich, Germany,; 3 German Center for Infection Research (DZIF), Partner Site Munich, Munich, Bavaria, Germany,; 4 Chantal BIYA International Reference Centre for Research on HIV/AIDS Prevention and Management (CIRCB), Yaoundé, Cameroon,; 5 Department of Biochemistry, Faculty of Science, University of Bamenda, Bambili, Bamenda, Cameroon,; 6 Division of Hepatology, Department of Medicine II, Leipzig University Medical Center, Leipzig, Germany,; 7 BIOMEX GmbH, Heidelberg, Germany,; 8 National Veterinary Laboratory, Garoua, Cameroon,; 9 Department of Pharmacotoxicology and Pharmacokinetics, Faculty of Medicine and Biomedical Sciences, University of Yaoundé I, Yaoundé, Cameroon,; 10 Faculty of Health Sciences, University of Buea, Buea, Cameroon,; 11 Faculty of Medicine and Biomedical Sciences, University of Yaoundé I, Yaoundé, Cameroon

**Keywords:** HIV drug resistance, Cameroon, viral load, antiretroviral therapy, rural health

## Abstract

**Introduction:**

despite the success of antiretroviral therapy (ART), the emergence of HIV drug resistance (HIVDR) remains a major threat in sub-Saharan Africa, where therapeutic options remain limited. With the goal of supporting ART response, we sought to monitor viral load (VL) response and acquired HIVDR emergence among patients initiating ART in the Cameroonian setting.

**Methods:**

a facility-based cohort study was conducted from March 2016 to May 2021 in urban (Yaoundé) and rural (Obala) settings in the Centre Region of Cameroon. Included were recently diagnosed HIV individuals initiating ART at the level of the health facilities. VL was measured at three different time points. For those with unsuppressed viremia (>1000 copies/mL), genotyping for HIVDR was performed in the protease, reverse-transcriptase, and integrase gene regions, and interpreted using HIVdb.v9.1. Data were analyzed with p<0.05 considered significant. Time-to-event analysis (Kaplan-Meier and Cox regression) was used to identify determinants of virological failure.

**Results:**

overall, 87 newly diagnosed participants (50.6% from urban and 49.4% from rural) were enrolled. Median (interquartile range, IQR) age was 42 (34.0-50.5) years, sex ratio (F/M) was 3/2, and all participants initiated treatment with non-nucleoside reverse transcriptase inhibitor (NNRTI)-based ART regimen. At initiation, median VL was 34,000 (13,963-122,000) copies/mL; at T1 (~3 years after initiation), median VL dropped to 9,800 (4,700-30,500) copies/mL, and 17.2% (15/87) switched to protease inhibitor-based ART. At the end of the study (T2), 58.6% (51/87) had achieved undetectable VL (<40copies/mL), 3.4% had VL between 40-999 copies/mL, and 37.9% VL >1000 copies/mL. The proportion with virological failure was 9.1% (4/44) in the urban setting versus 67.4% (29/43) in the rural setting. Time-to-event analysis revealed that patients in the rural setting had a 4.6-fold higher risk of virological failure (hazard ratio (HR) = 4.60, 95% CI: 2.29-9.27). Among those with unsuppressed VL, overall rate of HIVDR was 62.5% (20/32), driven by the mutations: M184V (31.25%) for NRTI, K103N (18.75%) for NNRTI and M46I (9.30%) for PI/r, and 0% major resistance mutations to integrase strand transfer inhibitors (INSTI), without any significant disparity between urban and rural.

**Conclusion:**

viral load monitoring reveals poor ART response in rural settings, which prompts the need for improving access to ART. Among those with unsuppressed VL, the burden and patterns of HIVDR are similar in both settings, likely due to the wide use of NNRTI-based ART. Viral susceptibility to INSTIs supports a possible switch to dolutegravir-based ART for optimal response.

## Introduction

The global community continues to grapple with the HIV epidemic, with an estimated 39.0 million people living with the virus worldwide in 2022 [1]. Approximately 0.7% (ranging from 0.6% to 0.8%) of adults aged 15-49 globally are HIV-positive, though the epidemic's burden varies significantly across different countries and regions [1].

The introduction of highly active antiretroviral therapy (HAART), in line with the joint program of the United Nations on AIDS (UNAIDS) 95-95-95 treatment target and WHO recommendation to test and treat, has all significantly contributed to decreasing the rate of HIV-related morbidity and mortality [2,3]. However, this major stride is currently being threatened by the occurrence of HIV drug resistance (HIVDR), specifically in resource-limited settings (RLS) [2,4-6]. The primary objective of antiretroviral treatment is to sustain the virus at an undetectable level, typically defined as a viral load (VL) of <50 copies/mL [5,7,8]. Furthermore, contemporary evidence indicates a negligible or near-zero risk of HIV transmission when an individual's viral load is suppressed (≤1000 copies/mL) [7].

Conversely, HIVDR arises when the virus successfully replicates in the presence of one or more antiretrovirals (ARVs) [9,10]. This occurs due to mutations in the HIV genome that modify the target proteins of ARV drugs, thereby affecting their ability to block viral replication. HIVDR is categorized into several forms: (a) acquired drug resistance (ADR), which develops in patients experiencing ART failure; (b) transmitted drug resistance, observed in a newly infected, ART-naive patient; and (c) pre-treatment drug resistance (PDR), detected in patients starting ART, irrespective of previous ARV exposure [4,6,11]. To optimize the efficacy of treatment programs, the WHO strongly advocates for continuous monitoring of VL response and HIVDR surveillance. In Cameroon, about 93% of people living with HIV were on antiretroviral therapy (ART) in 2022, with a viral suppression rate of about 89.2% among those with recorded VL results [12]. Previous research highlighted that while drug dispensing practices are generally appropriate and VL acquisition is around 62% at the national level, adherence to the ART program, duration on the ART regimen, viral suppression, and viral load acquisition are better in urban versus rural settings, specifically among adolescents and children [2].

Motivated by these disparities, this study was undertaken to measure the virological response to ART over time, determine the patterns of drug resistance among viremic patients, and identify their determinants among patients initiating ART in urban and rural settings of the Centre Region, Cameroon.

## Methods

**Sample size and study design considerations:** the primary objective of this study was to conduct detailed virological monitoring and characterize acquired drug resistance patterns among patients initiating ART in urban and rural settings of Cameroon. This observational cohort study, designed for intensive laboratory follow-up, employed a convenience sampling approach, enrolling all eligible, consenting patients from Yaoundé Central Hospital (urban) and Obala District Hospital (rural) during the study period. The study was not powered a priori for a specific effect size for predictors of ART failure, as its primary aims were descriptive. The sample size of 87 participants reflects the cohort available for complete follow-up with the requisite laboratory data.

**Study setting and population:** this study was conducted in Cameroon, a Central African country at two sites: Yaoundé Central Hospital (urban) and Obala District Hospital (rural). Site selection followed community mapping conducted during previous HIV/AIDS intervention programs, with active participation from center coordinators, psychosocial workers, and community relay agents. Yaoundé Central Hospital, a large urban center established in 1998, provides comprehensive HIV treatment and care. As of 2023, it serves 10,000-12,000 of its 40,000 PLHIV cohort with ART, utilizing a substantial staff including physicians, nurses, laboratory technicians, social workers, and support staff. Obala District Hospital, a rural facility established in 2005, serves a smaller cohort of 2,757 PLHIV receiving ART out of a total of 2,998 PLHIV. Its staffing complement, also as of 2023, reflects its smaller scale. Sampling was carried out from March 2016 to February 2021.

Participants were newly diagnosed with HIV-1 infection, aged 21 years and above, and ART-naive. The following exclusion criteria were applied: a history of prior ART, re-initiation of ART after a treatment interruption, unsuccessful sample amplification or sequencing, co-infection with hepatitis B or C, and loss to follow-up (defined as defaulting on study visits). Patients co-infected with hepatitis were excluded to limit the potential confounding effects of medications used to treat hepatitis B (tenofovir, lamivudine, emtricitabine) on the selection of primary HIV drug resistance (PDR) [13]; hepatitis C virus (HCV)/HIV co-infection also exhibits differences in viral genotypes as compared to mono-infection [14].

**Variable:** this study investigated the impact of location (urban vs. rural) and time (baseline, ~3 years post-ART initiation, and end of study) on viral load (VL) and HIV drug resistance (HIVDR) among newly diagnosed HIV-1-infected adults initiating ART. VL, categorized as undetectable (<40 copies/mL), detectable (40-999 copies/mL), or unsuppressed (>1000 copies/mL), served as a primary outcome, reflecting ART response. HIVDR, including overall presence/absence, resistance to specific drug classes (NRTI, NNRTI, PI, INSTI), and specific mutations (M184V, K103N, M46I), was another key outcome. Other variables included age, sex, and ART regimen. The study aimed to determine if location influenced VL suppression and the development of drug resistance over time.

### Laboratory assessments

**Blood collection:** following voluntary informed consent, a venous blood sample was collected from each eligible participant.

**Viral load measurement:** viral load (VL) measurement was performed at three distinct time points: at the time of enrollment (baseline), during follow-up visits at the respective local study sites in Cameroon, and at the study's conclusion. While initial and follow-up VL assessments were conducted locally, all final VL testing was centralized and performed at the Max von Pettenkofer Institute's Virology Department. This centralized testing ensured consistency and quality control. The institute's accredited routine diagnostic procedures were followed, utilizing the Abbott m2000sp/rt system and the Abbott RealTime HIV-1 assay (art. no. 02G31-010). All assays were performed strictly according to the manufacturer's instructions to maintain the accuracy and reliability of the VL results.

**RNA extraction, amplification, and sequencing:** RNA was extracted from plasma samples using the QIAamp Mini Kit (Qiagen, Courtaboeuf, France), adhering strictly to the manufacturer's recommended protocol. HIV-1 genotypic resistance testing (GRT) was performed on samples from patients exhibiting unsuppressed viremia, defined as viral load (VL) greater than 1000 copies/mL. For GRT, HIV-1 RNA extracted from patient plasma was first reverse transcribed into cDNA using an HIV-1-specific primer. Target gene regions within the *pol* gene, encompassing the protease, partial reverse transcriptase, and integrase coding regions, were then amplified using nested polymerase chain reaction (PCR). The PCR products were subsequently sent to an external service provider for Sanger sequencing. To confirm subtype and rule out any potential cross-contamination between samples, a phylogenetic tree was constructed using the maximum likelihood method implemented in MEGA software version 7.0.26. HIV-1 drug resistance mutations (DRMs) were identified and interpreted using the Stanford HIV Drug Resistance Database algorithm [15]. The analysis focused on clinically relevant resistance to the major classes of antiretroviral drugs: nucleoside reverse transcriptase inhibitors (NRTIs), non-nucleoside reverse transcriptase inhibitors (NNRTIs), protease inhibitors (PIs), and integrase strand transfer inhibitors (INSTIs). Any sample exhibiting a mutation, whether in pure mutant form or as a mixture of wild-type and mutant variants, at known key amino acid positions associated with drug resistance, was classified as harboring the respective resistance mutation.

**Statistical analysis:** data were processed using the Microsoft Excel spreadsheet and analyzed using Epi Info V7 (CDC, Atlanta, GA). The Chi-square test and Spearman's correlation were used where appropriate, with r ≥ 0.8 considered a strong positive correlation and p <0.05 considered statistically significant. Participants lost to follow-up (LTFU) were excluded from longitudinal genotypic resistance analysis due to missing specimens. To address potential bias from this exclusion, we performed complete-case and intention-to-treat (ITT) analyses for the primary virological outcome at study end (T2). The primary result is the complete-case analysis (available viral load data at T2). The ITT analysis conservatively classified all participants LTFU after baseline (T0) as having virological failure (VL >1000 copies/mL) to estimate a worst-case virological failure rate. A time-to-event analysis compared virological failure risk (first viral load >1000 copies/mL after ART initiation) between urban and rural settings. Participants remaining suppressed were censored. The Kaplan-Meier method and log-rank test were used to estimate survival curves and compare groups. Hazard ratio (HR) with 95% confidence intervals (CI) was calculated. Multivariable Cox regression identified independent determinants of virological failure, adjusting for age and gender. The model included health facility location (urban/rural).

**Ethical consideration:** ethical approval for this research was obtained from the Cameroon National Ethics Committee for Research on Human Health (Ref. N° 2016/03/727/CE/CNERSH/SP), along with administrative authorization from the Regional Delegate of Public Health of the Center Region. All patients provided informed written consent for participation and sample storage, with adults giving consent and minors providing assent. Participation was voluntary, and individuals could withdraw at any time without penalty. All participants were treated equally, regardless of social status, gender, or other factors. Confidentiality and privacy were ensured through unique identifiers. Genotypic results were shared with participants to enhance their understanding of their viral profiles and tailor their treatment management.

## Results

**Socio-demographic data analyses:** from a total of more than 5000 active patient records across the study sites, 87 patients initiating antiretroviral therapy were successfully followed. Of these, 44 (50.6%) were from the rural site and 43 (49.4%) were from the urban site. The median age of participants was 42 years (interquartile range (IQR) 34-50.5). This prospective cohort study (March 2016 to February 2021) monitored viral load response and the emergence of acquired HIV drug resistance in newly diagnosed, ART-naïve Cameroonians aged 21 years or older. Exclusion criteria included: prior ART exposure, re-initiation following a treatment interruption, unsuccessful sample amplification or sequencing, co-infection with hepatitis B or C, and loss to follow-up. Table 1 provides a comprehensive summary of the baseline socio-demographic and clinical characteristics. The data presented includes the frequency (n) and percentage (%) for sex, age group, marital status, mode of transmission, WHO clinical stage at enrollment, current therapeutic issues and complaints, and initial and final treatment protocols. These factors offer insights into variables potentially influencing VL suppression and HIVDR development during the study period.

**Table 1:** socio-demographic and clinical characteristics of 87 ART-naive HIV-1 infected adults at baseline enrolment in a prospective cohort study, Yaoundé Central Hospital (Urban) and Obala District Hospital (Rural), Cameroon

Parameter		Frequency (n=87)	Percentage (%)
Sex	Female	52	60
	Male	35	40
Age group	(21-35)	24	27.58
	(36-51)	41	47.12
	(52-69)	22	25.28
Marital status	Married	35	40.22
	Single	31	35.63
	Widowed	21	24.14
Presumed mode of contamination	Exposure to sharp objects	8	8.00
	Unknown	15	17.24
	Sexual	64	73.56
WHO clinical stages at enrolment	I	70	80.46
	II	0	0.0
	III	17	19.54
	IV	0	0.0
Reported therapeutic issues and challenges	Non-adherence	13	14.94
	Poor therapeutic education	15	17.24
	Other	8	9.20
	Drug supply challenges	14	16.09
	Social stigma	21	24.14
	None	16	18.39
Initial ART regimen	AZT+3TC+EFV	36	41.38
	TDF+3TC+EFV	24	27.59
	AZT+3TC+NVP	15	17.24
	TDF+3TC+NVP	12	13.79
ART regimen at study end	AZT+3TC+EFV	26	29.88
	TDF+3TC+EFV	23	26.44
	AZT+3TC+NVP	12	13.79
	TDF+3TC+NVP	11	12.64
	LPV/r+3TC+TDF	12	13.79
	ATV/r+3TC+TDF	2	2.30
	DRV/r+3TC+TDF	1	1.15

AZT: zidovudine; 3TC: lamivudine; EFV: efavirenz; TDF: tenofovir; NVP: nevirapine; LPV/r: lopinavir/ritonavir; ATV/r: atazanavir/ritonavir; DRV/r: darunavir/ritonavir; ART: antiretroviral therapy

The majority of participants were female (60%) and aged between 36 and 51 years (47.12%). Regarding marital status, the most frequent categories were married (40.22%) and single (35.63%), followed by widowed individuals (24.14%). The primary mode of HIV transmission was sexual contact (73.56%), with smaller proportions attributed to unknown routes (17.24%) and sharp object exposure (8.00%). At enrollment, most participants were classified as WHO clinical stage I (80.46%), indicating relatively early-stage infection. Commonly reported therapeutic issues and complaints included social stigma (24.14%), poor therapeutic education (17.24%), drug supply challenges (16.09%), non-adherence (14.94%), and other issues (9.20%), while some participants reported no issues (18.39%). The most frequently initiated ART regimens were AZT+3TC+EFV (41.38%) and TDF+3TC+EFV (27.59%). By the end of the study, while these two remained common, a notable proportion of participants had switched to PI-based regimens, primarily LPV/r+3TC+TDF (13.79%), indicating potential treatment changes due to various factors, including potential drug resistance or tolerability issues. This switch in therapy is an important observation and suggests that treatment regimens were adjusted over time.

**Figure 1:**
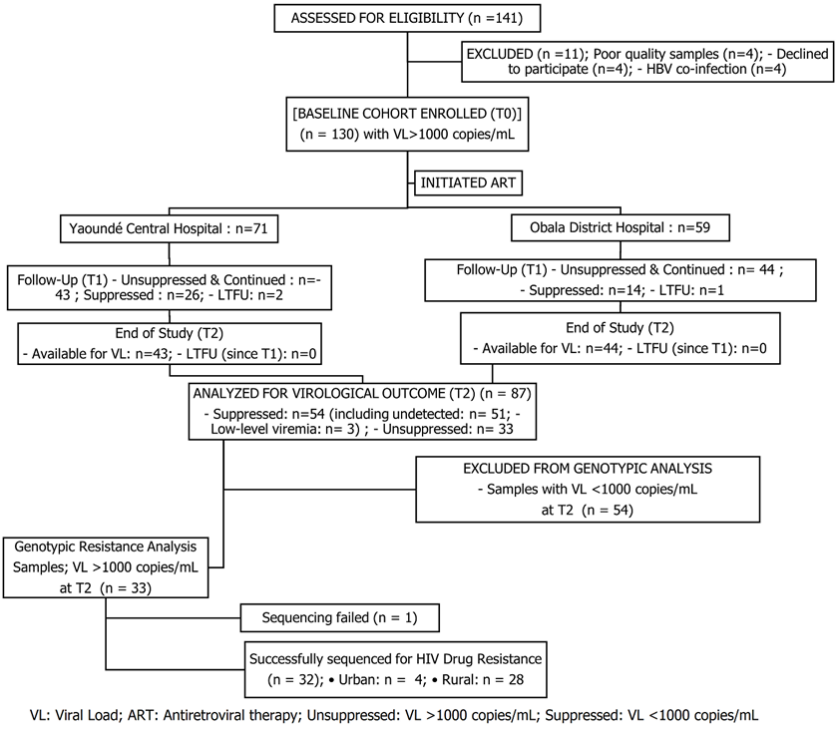
participant flow Diagram for the prospective cohort study monitoring virological response and acquired drug resistance in urban and rural Cameroon

**Participant flow and cohort retention:** the flow of participants through the study is detailed in Figure 1. From a source population of over 5,000 active patients across both sites, 141 patients initiating ART were assessed for eligibility. Of these, 130 participants with unsuppressed viremia (VL >1000 copies/mL) were enrolled into the baseline resistance monitoring cohort (T0), after excluding 11 individuals (4 with HBV co-infection, 3 with poor quality baseline samples, and 4 who withdrew consent). The baseline cohort comprised 73 participants from the urban site and 57 from the rural site.

At the first follow-up (T1, approximately 2 years post-ART), a pronounced disparity in initial treatment response was observed. In the urban setting, 29 of 73 participants (39.7%) achieved virological suppression and exited the monitoring arm, compared to only 10 of 57 (17.5%) in the rural setting. Consequently, 43 urban and 44 rural participants with unsuppressed viremia continued follow-up. Four participants were lost to follow-up between T0 and T1 (1 urban, 3 rural).

At the final study timepoint (T2, approximately 4 years post-ART), 87 participants remained for final analysis (43 urban, 44 rural). Viral load testing at T2 confirmed virological failure (VL >1000 copies/mL) in 33 participants (37.9% of the final cohort), who were eligible for genotypic resistance testing. This group consisted of 4 participants (9.1%) from the urban site and 29 (67.4%) from the rural site, underscoring the significant disparity in long-term ART outcomes. Sequencing was successful for 32 of these 33 samples (96.9%), forming the basis for the acquired drug resistance analysis.

**Changes in viral load over time following ART initiation in a Cameroonian cohort:** viral load (VL) measurements were conducted at three time points: at enrollment (T0, baseline), approximately three years after ART initiation (T1), and at the study's conclusion (T2). At baseline (T0), participants exhibited a high median viral load of 34,000 copies/mL (13,963-122,000), indicative of active viral replication. Approximately three years post-initiation (T1), the median viral load decreased substantially to 9,800 copies/mL (4,700-30,500), demonstrating a partial response to ART. At this time point, 17.2% of participants (15/87) had been switched to protease inhibitor (PI)-based regimens, likely due to factors such as virological failure or tolerability issues with their initial NNRTI-based regimen.

**Figure 2:**
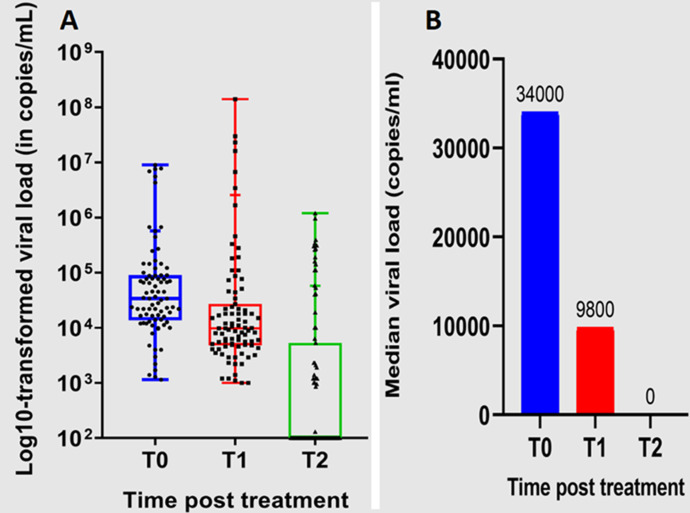
viral load dynamics within the study population at T0, T1 and T2: (A) viral load dynamics for all study participants over time; (B) variation in median viral load during the study

By the end of the study (T2), a significant proportion of participants, 58.6% (51/87), achieved virological suppression with undetectable viral loads (<40 copies/mL). An additional 3.4% (3/87) exhibited low-level viremia, defined as viral loads between 40 and 999 copies/mL. However, a substantial proportion, 37.9% (33/87), still had unsuppressed viral loads >1000 copies/mL at T2. Figure 2 (A,B) illustrates the dynamic changes in viral load observed among the participants throughout the study period. To account for attrition bias, an intention-to-treat (ITT) analysis was performed, which conservatively classifies the 3 participants lost to follow-up as having virological failure. In this ITT analysis (n=130), the proportion of participants with virological failure (VL >1000 copies/mL at T2 or LTFU) was 27.7% (36/130).

**Figure 3:**
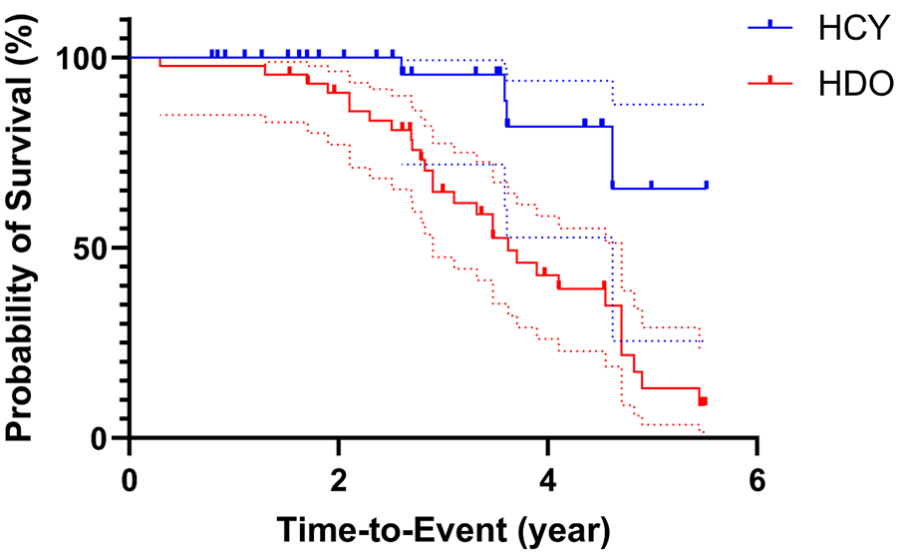
Kaplan-Meier curves for time to virological failure, by geographic setting

**Time to virological failure: urban-rural disparity:** the Kaplan-Meier survival analysis (Figure 3) was further used to compare the time to virological failure between participants from urban and rural settings. Kaplan-Meier analysis revealed a significant disparity in the time to virological failure between patients in urban and rural settings (p=0.0013, log-rank test; Figure 3). The median time to failure was 3.62 years for the rural cohort but was undefined for the urban cohort, as more than half of these patients remained virologically suppressed throughout the study period. The risk of virological failure was 4.6 times higher for rural participants compared to urban participants (hazard ratio (HR) = 4.60, 95% CI: 2.29-9.27). To control for potential confounders, a multivariable Cox regression analysis was performed (Table 2) to identify independent predictors of virological failure (defined as viral load >1000 copies/mL post-ART initiation). The model adjusted for health facility location, age, and gender. In this adjusted model, health facility location remained a strong and significant predictor of virological failure (aHR = 0.21, 95% CI: 0.06-0.53). This indicates that patients in the rural setting had a risk of virological failure that was 4.8 times higher (1/0.21) of virological failure compared to those in the urban setting, after controlling for age and gender. Neither age (aHR=0.98, 95% CI: 0.95-1.01) nor gender (aHR=1.15, 95% CI: 0.50-2.55) was significantly associated with the hazard of failure in this model (Table 2).

**Table 2:** cox proportional hazards regression analysis of predictors of virological failure among patients initiating antiretroviral therapy in urban and rural Cameroon

Variable	Adjusted hazard ratio (aHR)	95% confidence interval	P-value
Health facility (rural vs. urban)	0.21	0.06 - 0.53	<0.01
Age (per 1-year increase)	0.98	0.95 - 1.01	0.20
Gender (male vs. female)	1.15	0.50 - 2.55	0.73

Rural: District Hospital of Obala; urban: Central Hospital of Yaounde

**Figure 4:**
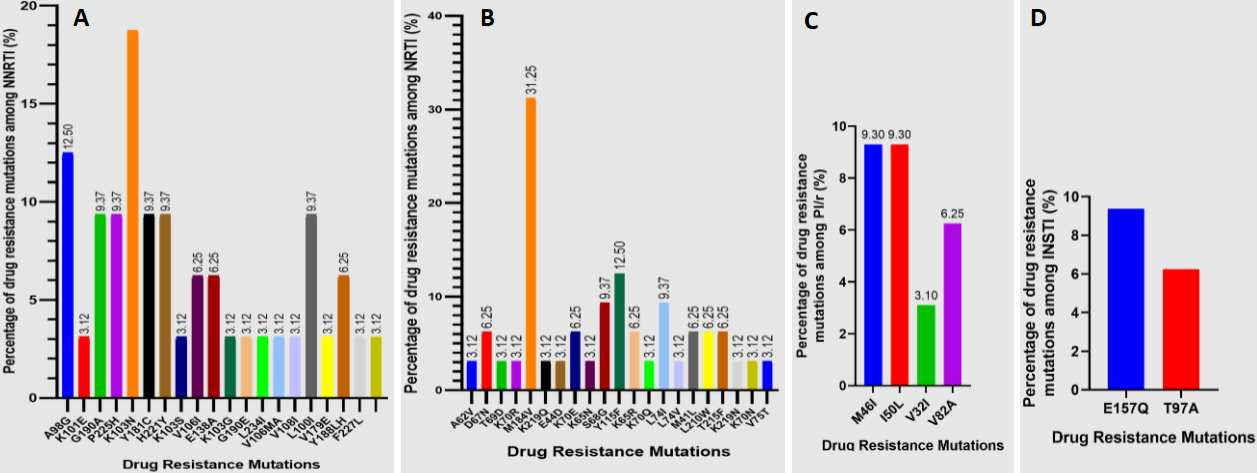
prevalence of drug resistance mutations by drug class: (A) NNRTI; (B) NRTI; (C) PI/r; (D) INSTI

**Drug resistance mutations in ART-failing patients in Cameroon:** of the 33 samples submitted for sequencing, successful sequences were obtained for 32 (96.9%), enabling a robust analysis of drug resistance mutations. The overall prevalence of HIV drug resistance (HIVDR) among these sequenced samples was 62.5% (20/32). While the prevalence of HIVDR was numerically higher in the urban setting (75%, 3/4) compared to the rural setting (60.71%, 17/28), this difference was not statistically significant. Figure 4 (A,B,C,D) presents the percentage of drug resistance mutations (DRMs) identified across different antiretroviral drug classes. Figure 4 A details NNRTI mutations, with K103N being the most prevalent at 18.75%, followed by A98G at 12.50%, and several other mutations present at lower frequencies. Figure 4 B, focusing on NRTI mutations, reveals a strong dominance of M184V at 31.25%, along with Y115F at 12.50% and a variety of other mutations at lower percentages. Figure 4 C depicts PI mutations, showing M46I and I50L both at 9.30%, with V82A and V32I present at lower levels. Finally, Figure 4 D illustrates INSTI mutations, where only E157Q and T97A are observed, at 9.37% and 6.25% respectively, and neither is considered a major INSTI resistance mutation. The figure highlights the differential prevalence of DRMs across drug classes, notably the high proportion of NRTI and NNRTI mutations, while PI mutations are less frequent, and major INSTI mutations are absent.

**Figure 5:**
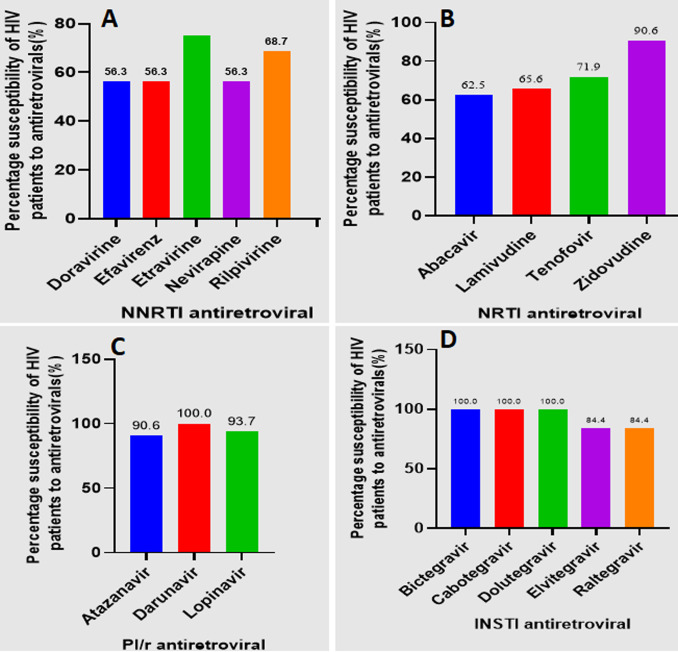
antiretroviral drug susceptibility by drug class: (A) NNRTI; (B) NRTI; (C) PI/r; (D) INSTI

**Antiretroviral susceptibility profiles:** Figure 5 (A,B,C,D) presents the susceptibility profiles of HIV to various antiretroviral drugs, categorized by drug class. Among NNRTIs, susceptibility varied. While 75% of patients (24/32) remained susceptible to etravirine and 68.7% (22/32) to rilpivirine, susceptibility to nevirapine, efavirenz, and doravirine was considerably lower, at 56.3% (18/32). For NRTIs, zidovudine maintained the highest susceptibility at 90.6% (29/32), followed by tenofovir at 71.9% (23/32), lamivudine at 65.6% (21/32), and abacavir at 62.5% (20/32). PI susceptibility was generally high, with 100% (32/32) susceptibility to darunavir, 93.7% (30/32) to lopinavir, and 90.6% (29/32) to atazanavir. INSTI susceptibility was notable, with 100% (32/32) susceptibility observed for bictegravir, cabotegravir, and dolutegravir, while elvitegravir and raltegravir showed slightly lower susceptibility, both at 84.37% (27/32). This figure highlights the varying levels of susceptibility across different ARV classes and individual drugs, demonstrating the importance of susceptibility testing to guide treatment decisions. Neither age, nor gender, nor treatment at initiation, nor viral load at initiation, was found to be associated with ART-failure. The only determinant of ART-failure was the rural area (p<0.00001; OR=20.7 (95%CI: 6.18-69.44)). Neither age, nor gender, nor geographic location, nor treatment at initiation, nor viral load at initiation, nor any other parameters were found to be associated with the emergence of HIVDR among these failing patients at the end of the study (all p>0.05).

**Figure 6:**
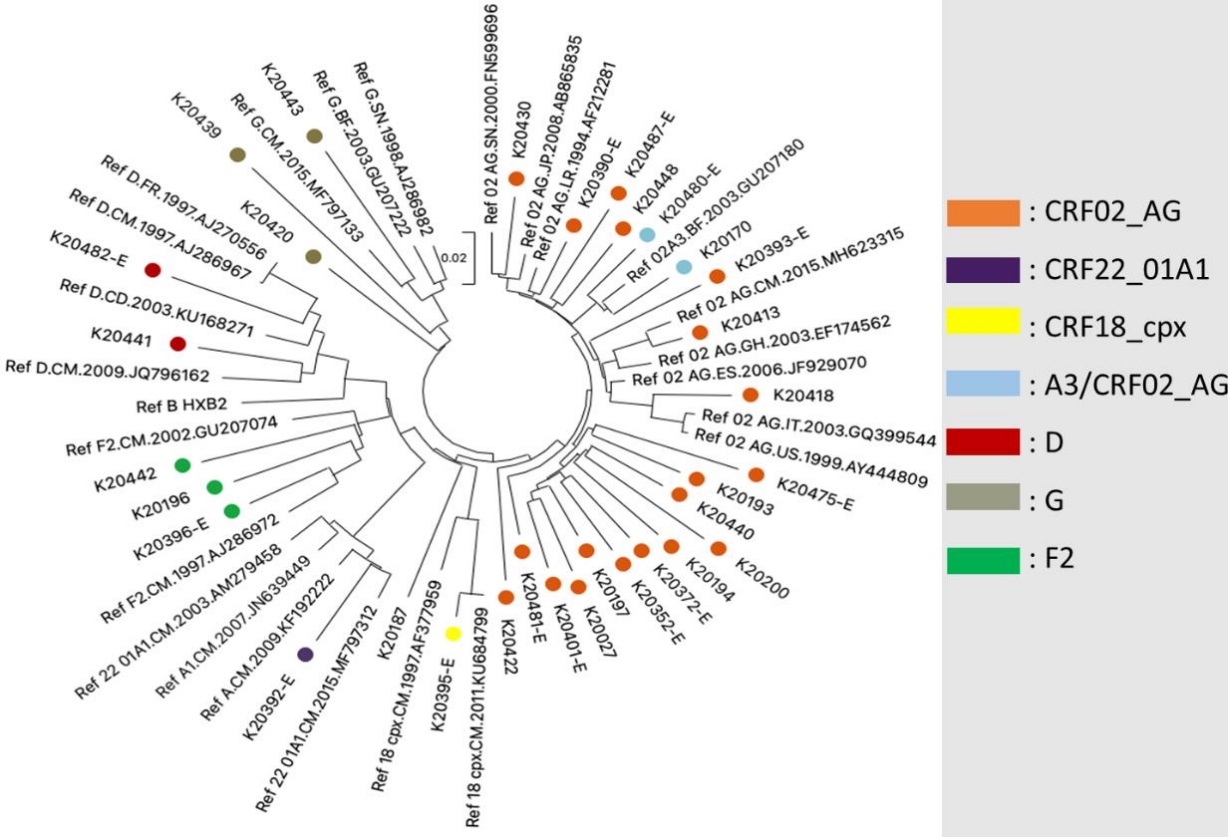
phylogenetic tree of protease and reverse transcriptase (PR/RT) sequences

**HIV genetic diversity:** seven HIV subtypes were identified in this study (Figure 6). CRF02_AG was the predominant subtype, accounting for 62.5% (20/32) of the sequenced samples, followed by subtypes F2 and G, each representing 9.4% (3/32) of the samples. Subtype D was found in 6.3% (2/32) of the samples, while CRF22_01A1, CRF18_cpx, and A3/CRF02_AG were each identified in a single sample (3.1%). This diverse distribution of HIV subtypes underscores the importance of genotypic testing for effective antiretroviral therapy.

## Discussion

The scale-up of highly active antiretroviral therapy has significantly reduced HIV-related morbidity and mortality through improved virological response. However, HIV drug resistance is rising, particularly in resource-limited settings (RLS) where ARV drugs with a low genetic barrier have been widely used [4-6]. Thus, exploring the dynamics of VL over time might help in depicting the appropriate timing for ART failure and in understanding the patterns of HIVDR selection over time for an optimal selection of ART regimens with long-term efficacy at the population level. Patients included in this cohort study were mostly middle-aged adults facing financial challenges, the majority of them being at an early stage of the infection, as revealed in our socio-demographic features. These results perfectly depict the socio-clinical context of HIV epidemics in most rural areas in RLS and are in line with the global data in Cameroon [12].

A central finding of our study is the profound disparity in ART outcomes between geographic settings. Using time-to-event analysis, we demonstrated that patients in the rural setting had a 4.6-fold higher risk of virological failure over time compared to their urban counterparts. This association remained strong and significant after adjusting for age and sex in a multivariable Cox model, indicating that rural residence is an independent determinant of poor treatment outcomes (Table 2).

As per our national guidelines at that time, all patients at enrolment were initiated on NNRTI-based first-line, and the switch was done after four years, following unsuppressed viremia. This result actually highlights the suboptimal prescribing practices in this cohort, which led to ART failure given the low genetic barrier of these drugs [16,17]. The significantly shorter median time to failure (3.62 years) in the rural cohort underscores the urgency of intervention. In contrast, although more than half of the population appeared to be controlling viral replication at the time, it remains crucial to shift to current guidelines using dolutegravir (DTG)-based treatment in order to ensure long-term success of ART among these patients. In effect, DTG is known to be highly effective in both treatment-naive and treatment-experienced individuals who may harbor multidrug resistance to other drug classes, with better drug tolerance, fewer drug interactions, higher potency, and a genetic barrier to resistance, both in vitro and in vivo, as supported by the recent WHO guidelines [5,18-22]. Therefore, implementing DTG-based ART will accelerate the achievement of viral suppression (i.e., VL <1000 copies/mL) in RLS, thereby reducing HIV transmission as promoted by the “undetectable=untransmissible” (U=U) campaigns [7,23].

Importantly, approximately one-third of the study participants experienced ART failure, and more than half of these patients harbored drug resistance mutations at the end of the study; rural setting was the only determinant of ART failure. Our longitudinal analysis confirms that the heightened risk in rural areas is not just a cross-sectional snapshot but a persistent disparity throughout the treatment journey. When employing a conservative intention-to-treat analysis that considered participants lost to follow-up as failures, the overall virological failure rate rose to 56.2%. This underscores the significant impact of attrition bias in cohort studies and suggests that programmatic ART failure rates may be substantially higher than those captured by standard monitoring of retained patients alone. Even though this result is unsurprising, it is worth noting that poor therapeutic education, non-adherence, and social stigma were prevailing among therapeutic issues and complaints that arose within this cohort of patients. Prevailing DRMs were M184V (for NRTI) and K103N (for NNRTI), in line with many other reports in similar settings [24-28]. Importantly, even though drug susceptibility at the end of this study was strongly in favor of current recommendations to switch to DTG-based in first-line (for patients who have achieved virological suppression/control) and in third-line (for patients failing PI-based regimens), high susceptibility to 3TC despite ART-failure following treatment with 3TC-containing regimens suggests poor adherence in this cohort [29]; thus calling for reinforced adherence support to prevent the rapid emergence of DRMs and ensure optimal therapeutic outcomes in the era of dolutegravir.

Finally, we identified seven different subtypes, with CRF02_AG being the most predominant, in line with HIV molecular epidemiology in Cameroon [8,30-34].

This study has several limitations that should be considered when interpreting the findings. First, the sample size was limited due to the use of convenience sampling, which may restrict the generalizability of the findings to the broader population. Second, the exclusion of participants lost to follow-up (LTFU) from the primary virological outcome analysis introduces a potential for attrition bias; as individuals lost to care are at a higher risk of virological failure, their exclusion likely leads to an overestimation of the true virological suppression rate. We have attempted to mitigate this by presenting a conservative intention-to-treat analysis, which provides a more programmatically relevant estimate of virological failure. Fourth, a shortage of CD4 count analysis supplies throughout the study period prevented CD4 monitoring of participants, limiting the assessment of immune status. Fifth, drug concentration monitoring was not feasible for patients with unsuppressed viral load to address potential non-adherence. However, the cohort monitoring system in place helped to partially mitigate the impact of non-adherence on study outcomes. Finally, the study was conducted prior to the national transition to TLD (tenofovir/lamivudine/dolutegravir) treatment. Viral load suppression rates might have been higher had the transition occurred earlier, given the observed full susceptibility of viral genotypes to INSTIs (integrase strand transfer inhibitors).

## Conclusion

In this Cameroonian setting, longitudinal monitoring revealed a significantly higher risk and shorter time to virological failure among patients in rural settings, which underscores the urgent need for targeted interventions to improve ART access and adherence support in these underserved areas. Among those with unsuppressed VL, HIVDR burden is high, driven predominantly by mutations associated with NNRTI-resistance both in urban and rural settings. Moreover, the mutational patterns indicate a low level of resistance to newer drug classes, such as PI/r, with no major resistance detected against INSTI. This high viral susceptibility to INSTIs supports the ongoing and necessary switch to dolutegravir-based ART for optimal response in both urban and rural settings within sub-Saharan Africa, sharing similar programmatic features with Cameroon.

### What is known about this topic

- Despite the success of antiretroviral therapy (ART), HIV drug resistance (HIVDR) remains a significant challenge, particularly in sub-Saharan Africa; limited therapeutic options in these settings make the emergence of resistance a major threat to effective treatment;
- Viral load (VL) monitoring is crucial for assessing ART response and identifying treatment failure, which can lead to the development of drug resistance; understanding VL trends and associated resistance patterns is essential for optimizing treatment strategies.

### What this study adds

- This study highlights a significant disparity in ART response between urban and rural settings in Cameroon, with poorer virological suppression observed in rural populations; this underscores the need for improved access to and adherence to ART in rural areas;
- The study provides data on the prevalence and patterns of HIVDR in Cameroon, specifically focusing on the distribution of resistance mutations in protease, reverse transcriptase, and integrase genes; the finding of high rates of NNRTI resistance and the absence of major INSTI resistance mutations supports the potential use of dolutegravir-based regimens in this population.
